# Urinary Sugars—A Biomarker of Total Sugars Intake

**DOI:** 10.3390/nu7075255

**Published:** 2015-07-15

**Authors:** Natasha Tasevska

**Affiliations:** Nutrition Program, School of Nutrition and Health Promotion, Arizona State University, 500 North 3rd Street, Phoenix, AZ 85004, USA; E-Mail: Natasha.Tasevska@asu.edu; Tel.: +1-602-827-2485; Fax: +1-602-827-2253

**Keywords:** sugars, diet, predictive biomarker, urine, sucrose, fructose, measurement error, validation, calibration

## Abstract

Measurement error in self-reported sugars intake may explain the lack of consistency in the epidemiologic evidence on the association between sugars and disease risk. This review describes the development and applications of a biomarker of sugars intake, informs its future use and recommends directions for future research. Recently, 24 h urinary sucrose and fructose were suggested as a predictive biomarker for total sugars intake, based on findings from three highly controlled feeding studies conducted in the United Kingdom. From this work, a calibration equation for the biomarker that provides an unbiased measure of sugars intake was generated that has since been used in two US-based studies with free-living individuals to assess measurement error in dietary self-reports and to develop regression calibration equations that could be used in future diet-disease analyses. Further applications of the biomarker include its use as a surrogate measure of intake in diet-disease association studies. Although this biomarker has great potential and exhibits favorable characteristics, available data come from a few controlled studies with limited sample sizes conducted in the UK. Larger feeding studies conducted in different populations are needed to further explore biomarker characteristics and stability of its biases, compare its performance, and generate a unique, or population-specific biomarker calibration equations to be applied in future studies. A validated sugars biomarker is critical for informed interpretation of sugars-disease association studies.

## 1. Introduction

Measurement error (ME) in self-reported diet has been a long-standing obstacle for determining the true association between sugars and chronic disease risk. While the evidence for sugars’ association with dental caries [1] and weight gain [2,3,4] has been rather consistent, the link to cardiovascular disease (CVD) [5,6,7,8], type 2 diabetes [9,10] and cancer [6,11,12,13,14,15] has been ambiguous and inconclusive. Although added sugars consumption in the United States (US) has declined over the last ten years, it still remains high, particularly among children (17% of energy intake [EI]) and young adults (16% EI) [16,17]. However, despite the high prevalence of this behavior and its implicated adverse effects on health, the inconsistency in evidence obtained from large prospective studies and randomized controlled trials has hindered the setting of a specific and unique recommendation on sugars intake in the US [18,19,20]. Sugars comprise monosaccharides (glucose, fructose, galactose) and disaccharides (sucrose, maltose, lactose), and their sum is known as “total sugars.” Sugars that are naturally occurring in fruits, vegetables, and dairy products only partly account for total sugars intake, whereas sugars from highly processed food and drinks, added during food processing and preparation or at the table, have become more significant contributors of total sugars intake [21]. As a source of empty calories and a common ingredient of unhealthy foods, sugars are among the nutrients that are frequently misreported [22,23]. It is highly plausible that ME in self-reported sugars may be obscuring the true relationship between sugars and disease risk and may explain the lack of consistency in the epidemiologic evidence. Development of novel approaches for obtaining more accurate estimates of intake, independent of self-reported diet, is crucial for attaining more valid and reliable risk estimates for sugars in relation to chronic disease risk. Until then, it is uncertain whether the lack of association is due to our inability to measure sugars accurately or to a genuine lack of an association between sugars and disease risk.

Dietary biomarkers hold a lot of promise as objective measures of intake in diet-related studies. Thus far, four categories have been described based on biomarkers’ characteristics: recovery, predictive, concentration, and replacement biomarkers [24]. They have multiple applications, including (1) in dietary validation studies, to characterize ME in dietary self-reports [25,26]; (2) in calibration studies nested within prospective cohorts, to develop calibration equations for “correcting” self-reported intake in the main studies [27,28,29]; (3) in population studies with available biological samples, as measures of dietary exposure, either alone [30,31,32,33] or in combination with self-reports [34,35]; and (4) in dietary intervention studies as measures of compliance [36].

## 2. Development of the 24 h Urinary Sucrose and Fructose Biomarker

### 2.1. Preliminary Work

Early work has shown that under physiological conditions, small amounts of dietary sucrose [37] and fructose [38] are excreted in the urine. During digestion, sucrase hydrolyzes sucrose into glucose and fructose in the brush border of the duodenum. Although healthy gastrointestinal mucosa is relatively impermeable to disaccharides, under physiological conditions, very small amounts of unhydrolyzed dietary sucrose may pass the intact intestinal wall, probably by a process of non-mediated diffusion [37], and once in the circulation, sucrose is readily excreted in urine [39]. Fructose is absorbed unchanged in the lower part of duodenum and jejunum; it is passively transported by a fructose-specific facilitative transporter (GLUT5) across the apical membrane of the intestinal epithelial cells and by a facilitative transporter for glucose and fructose (GLUT2) across the basolateral membrane into the circulation [40,41]. The amount of fructose occurring in urine is probably a fraction of the dietary fructose or fructose derived from sucrose that escapes the uptake by the liver, as the main site of fructose metabolism, and by other tissues, such as kidneys, adipose tissue and skeletal muscle [42,43], and that escapes the reabsorption in the renal tubules [41]. Considerable amounts of fructose were detected in urine after ingesting sucrose in a bolus [38]. Although some amount of glucose can be measured in urine, it appears to be person-specific and is not reflective of dietary intake due to insulin-controlled glucose reabsorption occurring in the kidneys.

The preliminary work on sucrose and fructose as potential biomarkers of sucrose intake was conducted by Luceri *et al.* [44], based on data from nine participants consuming a “regular Italian” diet for a week and a low-sucrose diet for three days. Sucrose and fructose were measured in spot urine samples collected within 2 h of breakfast, lunch, and dinner on the last day of each study diet. Sucrose and fructose excretion during the low-sucrose diet was significantly lower compared than the excretion during the regular diet. Furthermore, the sucrose content of both the low-sucrose and regular diet, as assessed by a food diary, was significantly associated with the post-meal urinary excretion of sucrose and fructose. As the slopes of the regression lines calculated from the regression models for the low-sucrose and regular diet were similar, a common slope for the association between sucrose intake from low-sucrose or regular diet and sucrose excretion was calculated (β = 0.26; SE = 0.08). Similarly, a common slope for the association between sucrose intake from low-sucrose or regular diet and fructose excretion was reported (β = 0.15; SE = 0.05). No correlation was found between the urinary excretion of glucose and intake of sucrose. The findings from this study implied that sucrose and fructose may have the potential to be used as biomarkers of sugars consumption, however further work was needed to study the characteristics of the biomarker and to develop prediction equations based on a daily diet and against true intake.

### 2.2. Development of the Urinary Sugars Biomarker under Controlled Conditions

Urinary sugars as potential sugars biomarkers were then rigorously investigated under highly controlled conditions in two feeding studies conducted in the United Kingdom [45]. All dietary intake in these studies was known, and multiple 24 h urine samples were collected and verified for completeness using the para-amino benzoic acid (PABA) test [46]. The first study was a 30-day randomized cross-over design study involving 12 healthy males aged 25–77 years. In randomized order, all participants consumed low (63 g), medium (143 g), and high (264 g) total sugars diet over three 10 day dietary periods, respectively; this level of intake corresponded to the lower and upper 2.5 percentiles and median total sugars intake for the adult UK population [47]. All foods consumed by the participants were prepared in a metabolic kitchen, and no foods or drinks obtained outside the metabolic suite were allowed to be consumed. On Days 4–7 during each 10-d dietary period, participants collected 24 h urine samples, which were analyzed for sucrose and fructose. Although the within-subject variability of sucrose and fructose excretion was rather high in these 12 participants at the same level of total sugars intake, the mean urinary sucrose and fructose increased across the increasing levels of sugars consumption over the three dietary periods, and there was a significant difference in the mean excretion of both sucrose (*p* < 0.001) and fructose (*p* < 0.001) between the three diets. Given that the dose-response association between the diet and sugars excretion improved after combining urinary sucrose and fructose, their sum was further investigated as a potential biomarker.

The 2nd feeding study assessed the performance of the biomarker in subjects consuming their usual diets over an extended period of time, *i.e.*, simulating normal dietary behavior under controlled conditions [45]. Seven male and six female participants aged 23–66 years consumed their habitual diet (previously assessed by four consecutive 7-day diet records), and collected 24 h urine samples daily over 30 days while residing in a metabolic suite. All the foods consumed by the participants were prepared in the metabolic kitchen, where the foods were weighed to the nearest gram and left-overs weighed out upon return. The group 30 day mean sum of the 24 h urinary sucrose and fructose (24uSF) was 98 mg/day (range 25.4–267.5 mg/day), which represented a very low proportion of their 30 day mean total sugars intake (approximately 0.05%), yet, the two were highly correlated (*r* = 0.84; *p* < 0.001). The 24uSF was also highly correlated to sucrose intake (*r* = 0.77; *p* = 0.002). In the linear regression of urinary to dietary sugars, true total sugars intake explained 72% of the variability in the sucrose and fructose excretion, revealing sugars intake as a strong determinant of sucrose and fructose excretion [45]. The mean correlation between 24uSF measured from single 24 h urine and the “usual” total sugars intake was 0.71 [48]. In this study, the 30 day mean 24uSF was significantly correlated to the 30 day mean intake of extrinsic sugars *(i.e.*, any sugars or syrups added during processing and preparation of foods and drinks or added at the table, including sugars from fruit juices and honey.) (*r* = 0.84; *p* < 0.001) but not intrinsic sugars (*i.e.*, any sugars from fruits and vegetables (excluding fruit juices) and cereal and cereal products (excluding breakfast cereals, biscuits, cakes, sweet buns, pies, flans, pastries, scones, and cereal-based puddings)) (*r* = 0.43; *p* = 0.144) [49]. Nonetheless, this study was not designed to investigate the performance of urinary sugars as dietary biomarkers of extrinsic *vs.* intrinsic sugars. The stronger correlation observed with extrinsic sugars may have been due to the fact that the intake of intrinsic sugars in these 13 subjects was lower and narrower in range (68 ± 23 (SD) g/day) than was their extrinsic sugars intake (123 ± 41 (SD) g/day).

To investigate the effect of body mass index (BMI) on the performance and validity of the biomarker, Joosen *et al.* [50] compared the biomarker’s response to diets providing 13%, 30%, and 50% energy from total sugars, in a randomized cross-over design under controlled conditions, in 10 normal-weight and nine obese participants living in a metabolic suite over 12 days. The excretion of both sucrose and fructose in urine increased to a similar degree in both the normal-weight and obese participants with the increase in total sugars intake; no significant interaction effect of BMI on urinary sucrose (*p* = 0.65) or fructose (*p* = 0.55) was observed. These findings have lent support to the application of this biomarker as a valid measure of intake in participants regardless of their BMI, as well as to investigations of the relationship between the consumption of sugars and the risk of obesity.

Given that the sugars biomarker exhibited a high correlation to “true” intake and was related to intake in a dose-response and time-sensitive manner, yet was recovered in a very low proportion in urine, 24uSF was categorized into a new class called “*predictive*” biomarkers [45]. Unlike recovery biomarkers, which are gold standard reference instruments, free of bias [51], predictive biomarkers contain a certain level of person-specific, intake-related, and covariate-related bias [52]. However, for these measures to qualify as predictive biomarkers, their biases should not explain a significant proportion of the variability in the biomarker, should be stable between individuals and across populations, and be estimable from a feeding study. Once those biases have been estimated, they can be applied to “correct” or “calibrate” the biomarker that can then serve as a reference instrument [52]. Such an equation for “calibrating” the 24uSF biomarker was generated from the 2nd UK feeding study [45,52]. The equation describes the association between the 24uSF and “true” total sugars intake, quantifies the biases associated with the 24uSF biomarker, and “calibrates” the biomarker to provide an unbiased measure of intake:

(1) $$\;M_{ij}^{*}=M_{ij}-1.67-0.02\times S_{i}+0.71\times A_{i}\;\left(Equation\;\left(1\right)\right);$$

where $\;M_{ij}^{*}$ is the log-transformed calibrated sugars biomarker, $M_{ij}$ is the log-transformed biomarker, $S_{i}$ is an indicator variable that equals 0 for men and 1 for women, and $A_{i}$ is the log-transformed age in years. The calibrated biomarker $M_{ij}^{*}$ satisfies the following *ME model for predictive biomarkers*: $M_{ij}^{*}=T_{i}+u_{M_{i}}+{\text{ε}}_{M_{ij}}$, where *T_i_* is the log-transformed true usual intake of total sugars, $u_{M_{i}}\;$ is a person-specific bias, and ${\text{ε}}_{M_{ij}}$ is a within-person random error [52]. Assuming that these biases are similar between individuals and across populations, this biomarker calibration equation can be applied in other studies with available 24 h urine collections to “calibrate” the sugars biomarker to be used as a reference instrument for total sugars intake. If spot urines, rather than 24 h urine collections, are available in population studies, the biomarker “calibration” equation cannot be applied to provide an unbiased measure of intake, however, the biomarker can be used as a correlate to intake, *i.e.*, concentration biomarker.

### 2.3. Investigation of Urinary Fructose as a Biomarker against Self-Report in Children

Johner *et al.* [53] investigated the use of urinary fructose as a biomarker of sugars intake among children in a subsample from the Dortmund Nutritional and Anthropometric Longitudinally Designed (DONALD) study. The analysis included 58 boys and 56 girls with mean age of 9.3 ± 0.8 and 7.9 ± 0.7 years, respectively, with available 24 h urinary fructose and self-reported diet data. Total sugars intake assessed by a three day weighed food record was significantly associated with fructose excretion from a single 24 h urine (*r* = 0.43; *p* < 0.001); each gram of total sugars intake was associated with a 0.9% increase in the amount of 24 h urinary fructose. The R^2^ for the observed association was low (R^2^ = 0.18), most probably due to the availability of a single-day urinary measurement, which would have introduced considerable random error in the biomarker, and to the use of a self-reported diet. Based on the authors’ preliminary data, in which a less consistent dose-response relationship between sucrose intake and excretion was found, urinary sucrose was omitted from the analysis [53]. In contrast to previous studies [44,45], no preservative was used to preserve the 24 h urine collection but the urine was only refrigerated, which may have led to sucrose hydrolysis and thus to unreliability of the sucrose data. The lower correlation for fructose excretion observed by Johner *et al.* [53] may also have been partly due to fructose degradation or uptake by bacteria in preservative-free urine, in case of noncompliance with the storage instructions of their 24 h urine collection protocol.

## 3. Application of the Urinary Sugars Biomarker in Validation and Calibration Studies with Free-Living Individuals

Acknowledging the impact of ME, researchers have started conducting dietary validation studies with available biomarkers to assess ME in dietary self-reports [25,54]. They have also begun incorporating calibration substudies within their cohorts to develop regression calibration equations [27,28,29] that could then be applied to calibrate self-reported intake, *i.e.*, predict unbiased intake in all cohort participants to obtain more reliable risk estimates in diet-disease analyses [55,56,57,58,59,60].

The urinary sugars biomarker has recently been applied in two US biomarker-based studies, the Observing Protein and Energy Nutrition (OPEN) study [52] and the Nutrition and Physical Activity Assessment Study (NPAAS) [61]. The OPEN study involved 484 participants aged 40–69 years recruited from Montgomery County, MD, in 1999 and 2000 [62]. The NPAAS is a biomarker study embedded in the Women’s Health Initiative (WHI) Observational Study (*n* = 93,676), involving 450 postmenopausal women aged 50–79 years at baseline, recruited from nine WHI centers between 2007 and 2009 [27]. Participants from both studies completed a food frequency questionnaire (FFQ) and at least two 24 h dietary recalls (24HR), and the NPAAS participants additionally completed a four-day food record (4DFR). The 24uSF biomarker and doubly-labeled water, a biomarker for energy intake, were measured in both studies, with an aim to investigate ME in self-reported total sugars (g/day) and total sugars density (g/1000 kcal). Given that the 24uSF biomarker has been found to contain a certain amount of bias that has been estimated in a feeding study, the biomarker was first “calibrated,” using the biomarker “calibration” equation (Equation(1)) previously described [52], to provide an unbiased measure of total sugars intake. These applications assume that the biases in the biomarker are stable across different populations. Once “calibrated,” the biomarker was applied to the OPEN and the NPAAS data as a reference instrument to assess the ME in self-report instruments by estimating the Attenuation Factor (AF) and the correlation between true and self-reported intake. AF represents the slope in the regression of true on reported intake and measures the attenuation (underestimation) of the disease risk due to ME in self-reported intake, where observed relative risk (RR) = true RR^AF^ [63]. AF can have values between 0 and 1; AF = 1 would indicate no attenuation, whereas as AF gets closer to 0, the extent of attenuation increases. Table 1 reports the AFs for the instruments used in the OPEN and NPAAS, and the observed disease RR associated with self-reported sugars measured with error when true RR = 2. For absolute sugars, the AF for the FFQ was lower (less favorable) than the AFs for the average of two or three 24HRs, and, in the OPEN study, for both the 24HR and FFQ, the AFs were lower in women than in men. For sugars density, the AF for the FFQ was somewhat lower than for the 24HR and was lowest (least favorable) for the 4DFR.

Another estimate of ME in dietary self-report is the correlation between true and self-reported intake, calculated based on the ME model parameters of the biomarker and self-reports [63]. The correlation between the true and self-reported total sugars density in the OPEN participants was 0.5 for the FFQ and a single 24HR, and 0.6 for the average of two 24HRs in men, and 0.2 and 0.3 in women respectively [52]. The correlation between the true and self-reported total sugars density in the NPAAS women was of a similar magnitude to the correlation observed in the OPEN women, and was 0.3 for the FFQ, a single 24HR, and the average of two 24HRs, 0.4 for average of three 24HRs, and 0.2 for the 4DFR [61]. Based on the ME estimates from these two studies, women misreported sugars consumption more than men, and even though all investigated self-reporting instruments were associated with substantial ME, the average of multiple 24HRs was found to perform the best [52,61].

**Table 1:** Attenuation factors (AF) for self-reported total sugars in the OPEN and NPAAS studies from a measurement error model with urinary sugars biomarker as a reference instrument and observed relative risk (RR) for true RR = 2 [52,61].

		OPEN				NPAAS		
		Men		Women		Women		
		FFQ	24HR ^†^	FFQ	24HR ^†^	FFQ	24HR ^‡^	4DFR
Total sugars (g/day)	AF	0.28	0.41	0.17	0.29	0.22	0.34	0.33
	Obs RR (true RR = 2)	1.21	1.33	1.13	1.22	1.16	1.27	1.26
Total sugars density (g/1000 kcal)	AF	0.39	0.41	0.33	0.35	0.48	0.57	0.32
	Obs RR (true RR = 2)	1.31	1.33	1.26	1.27	1.39	1.48	1.25

Obs—Observed; FFQ—Food frequency questionnaire; 24HR—24 h dietary recall; 4DFR—4 day food record; ^†^: The average of two 24HRs; ^‡^: The average of three 24HRs.

Next, using the 24uSF biomarker in the NPAAS, regression calibration equations for total sugars (g/day) and total sugars density (g/1000 kcal) were derived by regressing the “calibrated” 24uSF biomarker, *i.e.*, unbiased intake, on the FFQ-reported total sugars intake and the baseline characteristics, which were found to be significant predictors of unbiased intake [61]. These calibration equations also allow an investigation of various baseline characteristics, known to be associated with dietary misreporting, as potential predictors of true intake, given the ME in the self-report [27,28]. These regression calibration equations can be used to “correct” the FFQ sugars and sugars density intake for ME in all WHI participants in future WHI association studies of sugars and disease risk to obtain more reliable risk estimates. The derived equations are as follows: regression calibration equation for total sugars, g/day: log-true intake = 3.51 + 0.23 × log FFQ + 0.17 × log Age − 0.11 × log BMI − 0.64 × current smoker + 0.55 × < high school (HS) graduate + 0.30 × HS graduate + 0.16 × some college + 0.04 × square-root metabolic equivalents/week; regression calibration equation for total sugars density, g/1000 kcal: log-true density = 0.49 + 0.44 × log FFQ + 0.77 × log Age − 0.37 × log BMI − 0.56 × current smoker + 0.64 × < HS graduate + 0.43 × HS graduate + 0.18 × some college.

## 4. Application of the Urinary Sugars Biomarker as a Measure of Dietary Exposure in Diet-Disease Association Studies

The urinary sugars biomarker has so far been used as a surrogate measure of intake in two analyses of the Norfolk arm of the European Prospective Investigation into Cancer (EPIC) investigating the effect of sugars consumption on obesity risk [32,33]. Given only spot urine samples were collected in this study population, the biomarker calibration equation could not be applied to obtain unbiased estimates of total sugars intake. Therefore, the biomarker was used as a concentration biomarker.

The first analysis was a cross-sectional investigation involving 404 obese (BMI > 30 kg/m^2^) and 471 normal-weight (BMI < 25 kg/m^2^) participants from the EPIC-Norfolk aged 45–75 years [32]. Participants in the highest *vs.* lowest quintile for the biomarker (*i.e.*, the ratio of urinary sucrose to fructose measured in spot urine) were at 2.4 times higher risk of being obese (age- and gender-adjusted OR = 2.44, 95% CI = 1.54–3.86; *p*_trend_ < 0.001). The risk of obesity was also positively associated with sucrose measured in spot urine (OR_Q5 *vs.* Q1_ = 1.82, 95% CI = 1.26–2.85; *p*_trend_ = 0.02). A non-significant inverse association between sugars intake and obesity risk was observed when FFQ-measured total sugars (OR_Q5 *vs.* Q1_ = 0.89, 95% CI = 0.54–1.43; *p*_trend_ = 0.4) or the ratio of FFQ-measured sucrose to fructose (OR_Q5 *vs.* Q1_ = 0.77, 95% CI = 0.48–1.25; *p*_trend_ = 0.3) were used as measures of sugars intake. Given that creatinine was highly correlated with BMI, and could not be used to adjust for urine concentration, the authors used the ratio of sucrose to fructose in urine as a measure of intake.

In the second more recent analysis involving 1734 participants from the EPIC-Norfolk aged 39–77 years, the BMI measured at baseline and after 3 years of follow-up was positively associated with urinary sucrose from spot urine, and inversely associated with self-reported sucrose intake assessed by a seven day diet record (7DR), FFQ or 24HR collected at baseline [33]. Participants in the highest *vs.* lowest quintile of urinary sucrose were 1.54 times more likely to become overweight or obese (OR_Q5 *vs.* Q1_ = 1.54, 95% CI = 1.12–2.12; *p*_trend_ = 0.008), whereas those in the highest *vs.* lowest quintile of 7DR-based sucrose intake were at 44% lower risk of becoming overweight or obese (OR_Q5 *vs.* Q1_ = 0.56, 95% CI = 0.40–0.77; *p*_trend_ < 0.0001) after three years of follow-up. The authors used urinary sucrose per specific gravity to control for urine concentration, rather than the ratio of urinary sucrose to fructose, due to their having fewer available samples with both sucrose and fructose values within the acceptable analytical range. However, in sensitivity analyses in a reduced sample that used the sucrose to fructose ratio as a biomarker, all risk estimates remained virtually unchanged. Available evidence has consistently linked increased sugars consumption with an increase in obesity risk [2,3,4]. In this population, such an association was detected when using urinary sugars from spot urine as a concentration biomarker but not when using self-reported intake.

## 5. Summary and Future Research

Applying a sugars biomarker in future diet-disease association studies is crucial for detecting unbiased disease-risk estimates for sugars consumption. Biomarkers have been successful in revealing diet and disease associations that otherwise have been difficult to ascertain. Energy intake has been found to be associated with increased risk of breast cancer [58], all-cancer [56], CVD [57] and type 2 diabetes [60]; and protein intake with risk of type 2 diabetes [60] and frailty [64]; using biomarker-calibrated self-report estimates but not using un-calibrated instruments. When biomarkers are collected for all participants, they can also be used as surrogate measures of intake in relation to disease, either alone [30,31,32] or in combination with self-reports [34,35]. High sugars consumption was associated with statistically significant increased risk of obesity when the urinary sugars biomarker was used as a measure of dietary exposure [32,33], whereas no association [32] or inverse association [33] was found when using self-reported intake.

Although the 24uSF biomarker has been developed under highly controlled conditions, has great potential, and exhibits considerably favorable characteristics for a biomarker, available data come from only a few controlled studies with limited sample sizes (*n* = 12, *n* = 13, *n* = 19) [45,50]. Data from these feeding studies showed that the biomarker contains bias, which may originate from the between-subject variability in sucrose and fructose absorption, fructose uptake by individual tissues or reabsorption in the kidneys, and may be determined by genetic, dietary or lifestyle factors, or physiological or medical conditions. Some variability may be due to the analytical methods or issues related to urine collection, processing or storage. Increased sucrose excretion has long been used as a marker of altered gastrointestinal permeability [65,66], attributed to either structurally damaged or inflamed mucosa occurring in gastroduodenal, celiac [67] or inflammatory bowel disease [68], or the presence of congenital sucrose-isomaltose deficiency, which is considered to be particularly rare [69,70]. The intake of certain medications (e.g., non-steroid anti-inflammatory drugs [NSAID] [71], proton pump inhibitors [72]) and alcohol have also been associated with increased gastrointestinal permeability [73], while smoking has been found to decrease permeability, and to reduce the adverse effect of NSAID and alcohol on the gastrointestinal mucosa during simultaneous exposure, possibly by protecting the intercellular tight junctions of the epithelium [73,74]. Intestinal inflammation and infection leads to decreased expression of GLUT5, a fructose-specific transporter in the brush border membrane, and thus may impair fructose absorption [41]. Although, GLUT5 is acutely and efficiently upregulated by fructose concentration in the intestinal lumen and facilitates absorption, fructose malabsorption has been shown to occur among healthy individuals at fructose doses higher than 15 g, particularly in presence of low luminal glucose concentration, as glucose significantly improves fructose transport through enterocyte [41,75].

In the applications of the biomarker “calibration” equation in the US-based studies [52,61], an assumption was made that the biomarker’s biases are stable across participants and across different populations, which may not hold and needs to be further investigated. The original feeding study, the source of the urinary sugars “calibration” equation, was conducted in the UK [45]. Total sugars composition in the UK diet differs from sugars composition in the diet in the US and other countries. In the UK, table sugar or disaccharide sucrose is the main caloric sweetener, while in the US, monosaccharide sweeteners (e.g., fructose, glucose, dextrose) derived primarily from corn represent more than half of the caloric sweeteners used in the food supply [76,77]. A significantly different sucrose to fructose ratio in the US diet may result in different levels of sucrose and fructose measured in urine. The presence of glucose in the intestinal lumen enhances fructose absorption [75]. Hence, consuming fructose as part of sucrose or from a diet with a similar content of glucose may result in higher fructose absorption and thus excretion. Therefore, the performance and applicability of the calibration equation for the 24uSF developed with the UK diet requires further investigation and validation among US participants. We need further highly-controlled studies conducted in different populations across geographical regions to further explore biomarker characteristics and the stability of its biases, compare its performance, and validate the existing calibration equation for the 24uSF, or, if more applicable, generate population-specific biomarker calibration equations to be applied in future studies. Obtaining large sample sizes (*n* > 100) in future controlled studies will be crucial for achieving precision in estimating the biases arising from between-subject variability and for reliably investigating their effect on biomarker performance (personal communications, L. Freedman). Preparing and analyzing duplicate diets in such studies will avoid introducing ME from food composition tables in the estimate of “true intake” and will further increase the precision of the biomarker calibration equations. Although ensuring completeness of urine collections may not be necessary in population-based biomarker studies [78], it is essential in controlled feeding studies. Misestimation of the 24 h urinary biomarker excretion due to incomplete urine collections may affect the biomarker calibration equation and thus may lead to errors in the estimation of unbiased sugars intake in future biomarker studies. Preserving 24 h urine during collection is required for maintaining the stability of the sucrose and fructose [48], and boric acid in a concentration of ≤2 g/L has so far been used [45]. Among the analytical methods applied to measure sucrose and fructose in urine, liquid chromatography and gas chromatography with mass spectrometry (LC/MS and GC/MS) techniques use low-cost consumables, however, require expensive instrumentation and technical expertise [79]. In addition, for the GC and GC/MS analytical approaches, the sample-preparation step is labor intensive and time consuming [79,80], whereas the colorimetric method can be easily set up in most laboratories and is compatible with boric acid as a preservative [45].

Due to large participant burden, complex logistics, and high costs, collection of 24 h urine samples is not always feasible in large population studies. Investigation of the utility of spot urines in assessment of sugars intake is much needed and would undoubtedly lead to findings that could have major practical implications for epidemiologic studies. The urinary sucrose and fructose biomarker is a short-term measure of intake excreted 2–6 h after ingestion [44], and so when measured in spot urine, the biomarker (besides its inherent biases) will also contain a certain amount of ME, depending on the timing of the spot urine collection relative to intake. In the scenario of a population study, these errors will be expected to attenuate the association between true intake and the biomarker and to make it unstable. Hence, when measured in spot urine, this biomarker may possibly be used as a concentration, rather than as a predictive biomarker. Further investigation under controlled conditions, and identification of determinants of errors associated with the sugars biomarker measured in spot urine, will inform its future applications as an unbiased instrument. The concentration of sucrose and fructose in partial collections has been shown to correlate well with total sugars intake [44] and has been used previously as surrogate measures of intake, showing a positive association with obesity risk in a population study [32,33], consistent with the current evidence [2,3,4].

Recently, the carbon stable isotope ratio (^13^C/^12^C, expressed as δ^13^C) was proposed as a biomarker of sugars intake in populations consuming sugars abundant with ^13^C, such as corn-based sugars and sugar cane [81,82,83]. Although measuring component-specific δ^13^C, such as δ^13^C in red blood cell alanine, has shown much promise [84], this biomarker has never been investigated under highly-controlled conditions of a feeding study. Investigating the comparative performance of the urinary sugars biomarker and δ^13^C in relevant populations under controlled conditions would be particularly useful. Furthermore, a panel of four urinary biomarkers (formate, citrulline, taurine and isocitrate) indicative of sugar-sweetened beverage consumption has been identified using a metabolomics-based approach, and further work needs to be conducted to define their use in population-based research [85].

The urinary sugars biomarker has so far been used in observational studies only. One of the critical limitations of intervention studies investigating the effect of sugars consumption has been the reliance on self-reported measures [5], thus application of the sugars biomarker as a measure of intervention compliance will inform the interpretation of findings and help obtain valid estimates of the intervention effect.

Methodologically rigorous development of the urinary sugars biomarker and its applications in population-based studies has shown that this biomarker exhibits favorable characteristics for a biomarker and has great potential, yet further investigation is needed to better characterize and inform its application in different populations. A validated sugars biomarker that can be applied in available and future observational and intervention studies with biological samples will serve as an instrumental resource that would allow correction for ME in self-reported sugars and detection of unbiased sugars-disease associations. Until strong and consistent evidence for adverse health effects of sugars is found, no firm advice can be given to the general public.
